# Histopathological characterisation of omphalitis in piglets

**DOI:** 10.1186/s13028-025-00826-5

**Published:** 2025-08-28

**Authors:** Sophie Amalie Blirup-Plum, Henrik Elvang Jensen, Søren Saxmose Nielsen, Katrine Top Hartmann, Mette Sif Hansen, Ken Steen Pedersen, Inge Larsen, Jens Peter Nielsen, John Elmerdahl Olsen, Egle Kudirkiene, Kristiane Barington

**Affiliations:** 1 Department of Veterinary and Animal Sciences, Faculty of Health and Medical Sciences, University of Copenhagen, Grønnegårdsvej 7, Frederiksberg C, DK-1870 Denmark; 2 Ø-Vet A/S, Køberupvej 33, Naestved, DK-4700 Denmark; 3 Statens Serum Institut, Bakterier, parasitter og svampe / Fødevarebårne Infektioner, Artillerivej 5, Kbh. S, DK-2300 Denmark

**Keywords:** Histology, Microbiology, Pathology, Piglets, Umbilicus

## Abstract

**Background:**

Antibiotic treatment of piglets after birth is commonly carried out due to concern for development of omphalitis leading to umbilical outpouchings and/or systemic infections. Among others, the portal of entry for bacterial infections includes the umbilical cord at birth. The aim was to characterise the histological and bacteriological pattern of manifestations in the umbilicus of piglets with omphalitis that died during the suckling period in a Danish herd.

**Results:**

A total of 37 piglets found dead or euthanised due to sickness before weaning were included. Histopathological omphalitis was diagnosed in 13 of these piglets, and umbilical lesions and bacteria were most often observed in association with the umbilical blood vessels. Neutrophilic granulocyte infiltrations were observed in association with both umbilical arteries and the vein, occurring most frequently in the arteries. *Escherichia coli* and *Trueperella pyogenes* were the most commonly isolated bacteria from piglets with histopathological omphalitis.

**Conclusions:**

Omphalitis in piglets was characterized by inflammation and presence of bacteria in the umbilical arteries and to a lesser extent the umbilical vein. Inflammation in urachus was not present.

## Background

The pig’s umbilical cord contains two umbilical arteries (*aa. umbilicales*), the umbilical vein (*v. umbilicalis*), and a tube leading from the bladder (urachus), all embedded in a specialized mucous connective tissue entitled Wharton’s jelly. Wharton’s jelly provides structural support and protects the umbilical vessels and urachus from compression. It contains delicate collagen fibres and cells containing gelatine-like mucus that encloses fibres that create an elastic effect [1, 2]. The urachus is a fibrous remnant of the allantois that transports urine from the bladder to the allantoic cavity. Histologically, the urachus consists of an innermost layer of modified transitional epithelium, similar to that of urothelium, followed by submucosal connective tissue, a layer of fibrous connective tissue, and an outer layer of smooth muscle cells [1]. After rupture of the umbilical cord, the umbilical arteries, the umbilical vein, and urachus will obliterate and eventually become remnants of fibrous connective tissue.

The rupture of the umbilical cord provides a potential portal of entry for pathogenic bacteria [3, 4], which may lead to severe infections within few days [4, 5]. Furthermore, omphalitis is assumed to contribute to the development of umbilical outpouchings, including umbilical hernia, cysts and abscesses, a common diagnosis in Danish pig herds [6]. However, in a recent study it was shown that it is not feasible to clinically diagnose infectious omphalitis in piglets of zero to three days of age [4], despite being a mandatory prerequisite for antibiotic use. Clearly, improved diagnostics of omphalitis are needed. However, before this can be achieved, it is essential to understand the progression and underlying causes.

The histological pattern of inflammatory manifestations in porcine omphalitis has not been described. Therefore, the aim of the present study was to conduct a histopathological characterisation of omphalitis in neonatal piglets by assessing the involvement of the key umbilical structures. In addition, the presence of bacteria in the umbilical tissues were evaluated by aerobic and anaerobic cultivation.

## Methods

### Herd and study design

The study was carried out in April 2021 in a Danish commercial indoor sow herd recorded in the Danish Central Husbandry Register [7]. The herd consisted of 850 sows and 4000 nursery pigs. Sows giving birth during three consecutive days were selected, and liveborn piglets of more than 600 g were ear tagged with a unique id number and included in the study. The piglets were followed till day 25 of life in the farrowing unit and all piglets found dead or euthanised due to illness were transported to the University of Copenhagen, where they were stored at 4 °C and subsequently sampled within 48 h.

### Sampling

Tissue samples from predefined areas of the umbilicus [4] were collected for histopathological examination. Swabs from the umbilicus were collected for microbiological culture as previously described [4].

### Bacteriological examinations

Swabs, placed in phosphate-buffered saline, were brought to the microbiological laboratory the same day and plated on two blood agar plates (blood agar base III; Oxoid ThermoFisher, Roskilde, Denmark) with 5% sterile calf blood, which were incubated for 18–20 h at 37 °C under aerobic and anaerobic conditions, respectively. Colonies of dominating phenotypes (pure culture or > 50% of colonies belonging to one type in mixed cultures) were purified on new blood agar plates, re-incubated under the same conditions as the primary plate, and then the species was identified by Matrix-assisted laser desorption ionisation time-of-flight (MALDI-TOF) mass spectrometry as previously described [8].

### Histopathology

The umbilical samples were fixed in 10% neutral buffered formalin for two-to-three days. Afterwards, the tissues were processed through graded concentrations of ethanol and xylene, and finally embedded in paraffin. Tissue sections of 4–5 μm were stained with haematoxylin and eosin.

The vessels of the umbilicus (*v. umbilicalis*,* a. umbilicalis sinister*,* a. umbilicalis dexter*), the urachus and Wharton’s jelly were examined. Blood vessels, which could not be identified due to lack of structures in the umbilicus were scored as ‘unidentified vessel’ and inflammation in these vessels as ’vasculitis’. For each structure in the umbilicus, scoring of manifestations was based on neutrophilic granulocyte (NG) infiltration as previously described [4]. In brief, a score of ≥ 41 NGs in one high power field (40x objective/0.75 numerical aperture) in any area was defined as histopathological omphalitis. Scores of ≤ 40 NGs were considered a part of normal umbilical involution. In each section, the areas with the highest density of NGs were selected for scoring. For the umbilical vessels specifically, the distinct layers of the vessel wall, i.e. tunica intima, media, and adventitia were scored separately. Finally, the inflammatory timing, i.e. acute or chronic, was determined for piglets with histopathological omphalitis. Chronic omphalitis was defined by the presence of granulation tissue in association with NG infiltration.

### Gram staining

Brown and Hopps gram staining for identifying and differentiating bacteria was performed on umbilical sections with a NG score of ≥ 40, i.e. the diagnostic threshold for histopathological omphalitis, as previously described [9].

## Results

### Study population

Born to 21 sows, a total of 37 piglets that died in the farrowing unit were included in this study. The age of the piglets varied from zero to 25 days, and 15/37 piglets were five days or older. Among the 37 piglets, 15 were females and 22 were males of which 10 were castrated.

### Histopathological findings

Of the 37 umbilical tissue sections, two were excluded due to autolysis (castrated males aged 18 and 23 days, respectively). In the remaining 35 sections, histopathology revealed omphalitis in 13 piglets of which six were female, four were intact males, and three were castrated males. The age of the piglets with histopathological omphalitis is demonstrated in Table 1.

**Table 1:** Location and distribution of lesions, age, and isolated bacterial pathogens in piglets with omphalitis (*n* = 13)

Pig No. (age, days)	Histology						Bacterial Pathogen	Antibiotic treatment
	Arteritis	Phlebitis	Vasculitis	Funisitis	Inflammatory timing	Gram stain		
1 (1)	Periarteritis, both arteries	Panphlebitis and periphlebitis	-	-	Acute	-	*S. suis*	No
2 (1)	-	-	-	Solely dispersed neutrophils	Acute	-	-	No
3 (2)	Periarteritis, both arteries	Periphlebitis	-	Solely dispersed neutrophils	Acute	-	-	Yes
4 (2)	-	-	Intravascular (tunica media)	-	Acute	Positive and negative	*E. coli*	No
5 (2)	Panarteritis, one artery	-	-	-	Chronic	-	-	No
6 (1)	Periarteritis, one artery	-	-	Microabscesses	Acute	-	-	No
7 (1)	Periarteritis, one artery	Periphlebitis	-	Microabscesses	Acute	-	-	No
8 (7)	-	Panphlebitis	Intravascular (tunica intima and media) and abscess formation	-	Chronic	Positive and negative	*E. coli*,* F. necrophorum*,* P. indolicus*,* T. pyogenes*	Yes
9 (4)	-	Periphlebitis	Intravascular (tunica intima and media) and abscess formation	Microabscesses	Chronic	Negative	-	No
10 (8)	Panarteritis one artery. Endarteritis and abscess formation, both arteries	-	-	-	Chronic	Positive	-	No
11 (22)	Endarteritis, panarteritis and abscess formation, both arteries. Periarteritis, one artery	-	-	-	Chronic	Positive	*T. pyogenes*	Yes
12 (24)	-	-	Intravascular (tunica intima and media) and abscess formation	-	Chronic	-	-	Yes
13 (25)	-	-	Perivascular	Solely dispersed neutrophils	Chronic	Negative	*E. coli*	Yes

Age, location and distribution of lesions in piglets with omphalitis based on histopathological evaluations, and identification of associated umbilical bacterial isolates. Bacterial identification was determined by culturing and matrix-assisted laser desorption ionisation time-of-flight. Piglets treated with antibiotics received Noromox Prolongatum Vet 150 mg/mL (15 mg/kg, Scanvet A/S, Fredensborg, Denmark) and/or Engemycin Vet 100 mg/mL (5-10 mg/kg, MSD Animal Health A/S, Copenhagen V, Denmark)

**Fig. 1:**
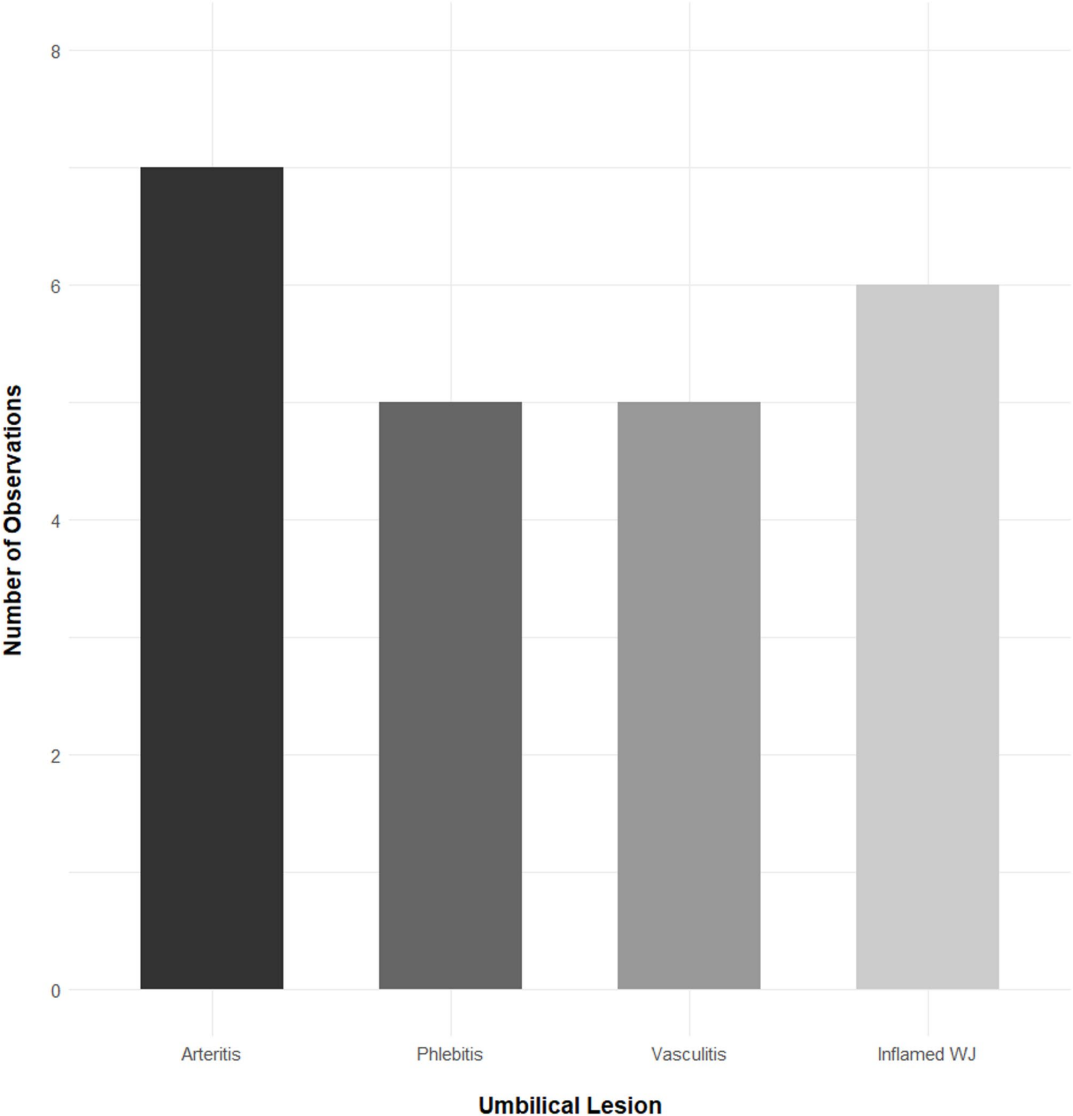
Number of histopathological lesions for the umbilical structures. A piglet was diagnosed with arteritis if signs of infection were present in one or both of the umbilical arteries. “Vasculitis” includes large vessels that could not be identified as either a vein or an artery. More than one lesion could be observed in some piglets. WJ; Wharton’s jelly

**Fig. 2:**
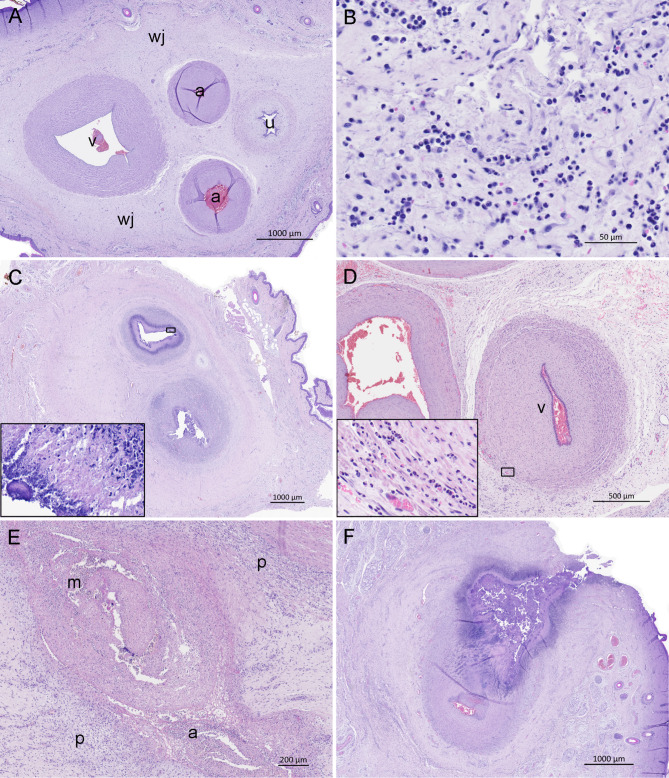
Scanned umbilical sections from piglets without (**A**) and with (**B**-**F**) histopathological omphalitis (haematoxylin and eosin stain, Zeiss Axioscan 7 microscope slide scanner, numerical aperture 0.8). (**A**) A two-day-old piglet with normal umbilical involution. Umbilical vein (v), arteries (a), urachus (u) and Wharton’s jelly (wj). Reused with permission from Blirup-Plum, 2024 [25]. (**B**) A two-day-old piglet with predominately neutrophilic granulocytes (NGs) and a few macrophages dispersed in Wharton’s jelly. (**C**) A 22-days-old piglet with arteritis in *a*. *umbilicalis* sinister and dexter. Infiltration with NGs in tunica intima and media, and necrosis of the tunica intima. Insert: Close-up of the necrosis. (**D**) A one-day-old piglet with NG infiltration of tunica adventitia and media in *v*. *umbilicalis* (v). Insert: Close-up of NG infiltration of tunica adventitia. (**E**) A four-day-old piglet exhibiting NG infiltration in the tunica intima, media (m), and adventitia (a), as well as perivascular (p) neutrophilic infiltration in an unidentified blood vessel within Wharton’s jelly. (**F**) A seven-days-old piglet with abscess formation in Wharton’s jelly. The manifestations consist of necrotic tissue demarked by NGs, macrophages and thrombus formation, and is affecting all structures of the umbilicus

The number of histopathological lesions for the various structures of the umbilicus are demonstrated in Fig. 1, and examples of lesions are shown in Fig. 2. In five of the 13 piglets, one or more of the essential umbilical structures (the umbilical vessels and urachus) had been obliterated and had become remnants of fibrous connective tissue.

The inflammatory infiltrate in piglets with histopathological omphalitis consisted predominately of NGs. However, mononuclear cells, especially macrophages, were also observed, with increased presence in chronic cases. In most of the piglets with histopathological omphalitis (12/13), bacteria and NG infiltration were seen in association with the vessels. In cases of arteritis and phlebitis, NG infiltration was most often found perivascular to the vessels (Table 1). However, intravascular NG infiltration, i.e. infiltration of tunica intima, media and/or adventitia, was also common. Abscess formation was observed in association with the umbilical arteries or unidentified vessels but not with the umbilical vein nor urachus (Table 1; Fig. 2F). The inflammatory timing (acute/chronic) of the manifestations are demonstrated in Table 1.

Only one piglet had NG infiltration solely in Wharton’s jelly (i.e. funisitis). No NG infiltration was seen in association with urachus in any of the piglets. However, haemorrhage and urolithiasis were observed in urachus in two piglets (both one day old) and a single piglet, respectively.

### Gram staining

**Fig. 3:**
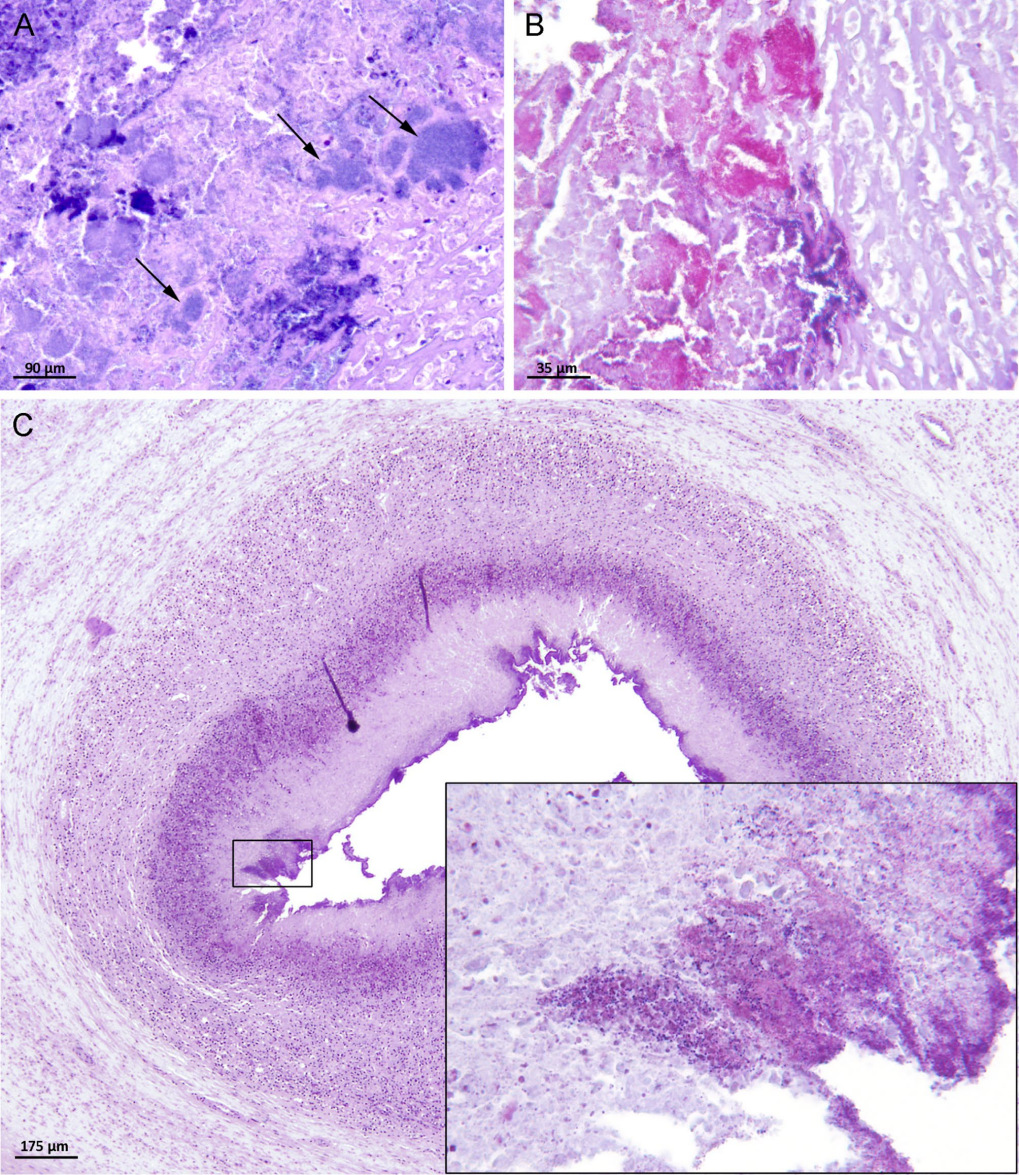
Umbilical sections from piglets with histopathological necrotising omphalitis (Olympus Orthoplan, DP72). **A** + **B**) A seven-day-old piglet with abscess formation in Wharton’s jelly (Fig. 2F). (**A**) Bacterial colonies (arrows) were observed near the centre of the abscess (haematoxylin and eosin stain, 40x/0.75). (**B**) Gram stain showing both gram-negative (pink) and gram-positive (dark violet) bacteria (40x/0.75). (**C**) A 22-day-old piglet with end- and panarteritis (gram stain, 25x/0.65). Insert: Coccoid, dark violet gram-positive bacteria infiltrating tunica intima and tunica media

Brown and Hopps gram-staining was performed on the 13 umbilical sections with histopathological omphalitis, and the results are demonstrated in Table 1; Fig. 3. Bacteria were observed in six piglets. In two piglets, both gram-negative and gram-positive bacteria were present. The remaining four comprised of two piglets with gram-positive and two with gram-negative bacterial infiltrations, respectively. (Table 1; Fig. 3).

### Bacteriological culture

Umbilical swabs revealed bacteria in five piglets with omphalitis (Table 1), with *E. coli* being the most common (3/13) followed by *T. pyogenes* (2/13).

## Discussion

Histological examination revealed omphalitis in 13/37 piglets that died before weaning. The occurrence of acute lesions in piglets aged 1–2 days and chronic lesions in those aged 2–25 days indicates that omphalitis develops soon after birth.

As in a previous study of omphalitis [4], the bacteria were histologically observed in association with the vessels with severe manifestations, making the piglets prone to haematogenous spread of bacteria and sequela such as arthritis, meningitis and umbilical hernia [3, 10, 11]. Another recent study has shown bacteria in the centre of abscesses in the umbilicus of piglets with omphalitis [12]. However, these piglets were 14 days old, and the walls of the umbilical vessels could no longer be identified by histological evaluation.

In the present study, inflammatory manifestations were observed in association with both the umbilical arteries and vein, occurring more often in the arteries. This is in accordance with another study on omphalitis in piglets [13]. The more frequent involvement of the arteries relative to the veins is somewhat counterintuitive, considering the arteries’ elevated blood pressure and flow velocity [14]. However, following umbilical rupture, the umbilical vessels and urachus retract into the abdominal cavity, initiating thrombosis and infiltration of inflammatory cells [1, 15], which subsequently may reduce the differences in pressure and velocity. One might also speculate that oxygen levels or nutritional content could play a contributing role, and further studies on bacterial affinity for the endothelial lining of veins and arteries could be interesting.

Reports on foals have shown omphaloarteritis to evolve and form abscesses in/near the terminal aorta and left ureter, thereby causing conditions such as haematuria, hydroureter and aortic aneurysm [11, 16]. In addition, studies on omphalophlebitis in cattle have demonstrated spread of infection to the liver leading to hepatic abscess formation [17, 18].

No signs of inflammation were observed in or adjacent to the urachus in any piglet, which is in accordance with other studies of humans and animals, where inflammation in the urachus were related to congenital abnormalities such as patent urachus and urachal cysts and diverticula [19, 20]. However, these results diverge from what is known for calves, in which the urachus seems to be the most frequently involved structure [21]. The urachus is a multilayered structure lined with transitional epithelium, resembling the urothelium [1, 22]. It features a highly specialized barrier that includes umbrella cells, a dense, gel-like physical barrier (glycocalyx), and immune components such as toll-like receptors and antimicrobial peptides, which makes it more resistant to bacterial invasion [22]. In contrast, pathogenic bacteria are skilled at manipulating and damaging the single layer of endothelium, e.g. by toxins that may disrupt cell-to-cell junction or kill the endothelial cells [23]. Thereby, the endothelium is considered more vulnerable to bacterial invasion than the urothelium, which may explain the differences observed in the inflammatory response in the present study.

Urolithiasis in one piglet appeared to be an incidental finding. Haemorrhage of the urachus was presumed to be associated with umbilical cord rupture, given that both piglets were only one day old.

*Escherichia coli* and *Trueperella pyogenes* were the most common bacteria isolated from the umbilicus of piglets with a diagnosis of histopathological omphalitis. This is in accordance with other studies of pigs and humans, where *E. coli* has been isolated from cases with omphalitis [4, 15]. In addition, *T. pyogenes*, often in combination with *E. coli*, has been reported to be amongst the most common causes of omphalitis and omphalophlebitis in ruminants [24]. No bacterial growth was detected in approximately half of the piglets diagnosed with omphalitis (6/13, Table 1). However, a limitation of the present study is that piglets might have received antibiotic treatment during the study period, which may obscure potential underlying infectious causes of omphalitis (Table 1).

## Conclusion

The present study provides a detailed histopathological assessment of omphalitis in piglets. Bacteria and lesions were identified in association with the blood vessels, and most often in association with arteries. This characterisation is essential to understand the underlying mechanism, which may help distinguish omphalitis from other conditions, e.g. oedema, dermal lesions, congenital malformations and umbilical herniation, and ensure proper management and treatment.

## Data Availability

The datasets used and/or analysed during the current study are available from the corresponding author on reasonable request.

## Abbreviations

MALDI-TOF: Matrix-assisted laser desorption ionisation time-of-flight
NG: Neutrophilic granulocyte
WJ: Wharton’s jelly
